# Calibrating a chief complaint list for low resource settings: a methodologic case study

**DOI:** 10.1186/s12245-021-00347-8

**Published:** 2021-05-19

**Authors:** B. Hansoti, E. Hahn, A. Rao, J. Harris, A. Jenson, N. Markadakis, S. Moonat, V. Osula, A. Pousson

**Affiliations:** 1 Department of Emergency Medicine, Johns Hopkins School of Medicine, Baltimore, MD USA; 2 Department of International Health, Johns Hopkins Bloomberg School of Public Health, Baltimore, MD USA

**Keywords:** Chief complaint, Emergency department, Symptom list

## Abstract

**Background:**

The chief or presenting complaint is the reason for seeking health care, often in the patient’s own words. In limited resource settings, a diagnosis-based approach to quantifying burden of disease is not possible, partly due to limited availability of an established lexicon or coding system. Our group worked with colleagues from the African Federation of Emergency Medicine building on the existing literature to create a pilot symptom list representing an attempt to standardize undifferentiated chief complaints in emergency and acute care settings. An ideal list for any setting is one that strikes a balance between ease of use and length, while covering the vast majority of diseases with enough detail to permit epidemiologic surveillance and make informed decisions about resource needs.

**Methods:**

This study was incorporated as a part of a larger prospective observational study on human immunodeficiency virus testing in Emergency Departments in South Africa. The pilot symptom list was used for chief complaint coding in three Emergency Departments. Data was collected on 3357 patients using paper case report forms. Chief complaint terms were reviewed by two study team members to determine the frequency of concordance between the coded chief complaint term and the selected symptom(s) from the pilot symptom list.

**Results:**

Overall, 3537 patients’ chief complaints were reviewed, of which 640 were identified as ‘potential mismatches.’ When considering the 191 confirmed mismatches (29.8%), the Delphi process identified 6 (3.1%) false mismatches and 185 (96.9%) true mismatches. Significant chief-complaint clustering was identified with 9 sets of complaints frequently selected together for the same patient. “Pain” was used 2076 times for 58.7% of all patients. A combination of user feedback and expert-panel modified Delphi analysis of mismatched complaints and clustered complaints resulted in several substantial changes to the pilot symptom list.

**Conclusions:**

This study presented a systematic methodology for calibrating a chief complaint list for the local context. Our revised list removed/reworded symptoms that frequently clustered together or were misinterpreted by health professionals. Recommendations for additions, modifications, and/or deletions from the pilot chief complaint list we believe will improve the functionality of the list in low resource environments.

## Introduction

The chief or presenting complaint is the reason for seeking health care, often in the patient’s own words. While the utility of recording and analyzing chief complaints is well accepted in resource-rich and highly developed emergency care systems, diagnosis-based research has remained the predominant standard when evaluating/quantifying burden of disease (BOD) in most health care settings [1]. In limited resource settings, a standard approach to quantifying BOD is not possible, partly due to both limited availability of an established lexicon or coding system and more limited diagnostics than in resource-rich settings [2]. Furthermore, diagnosis-based research strategies fail to capture an essential element of emergency care: the sorting of patients with undifferentiated symptom-based chief complaints into diagnostic categories and levels of acuity, which in turn guides decision-making on behalf of these patients based on limited symptom-based information and diagnostics.

The patient’s chief complaint is a key piece of information that helps direct this process. Additionally, the chief complaint can further be stratified based on acuity and those complaints designated as ‘high acuity’ have been found to independently predict mortality; enhanced attention and increased resources being made available to those with high risk chief complaints can improve patient health outcomes, and is the standard of care in many resource-rich settings [3]. This inherent value of the chief complaint has led to the development of ontologies of emergency care presenting complaints of varying degrees of sophistication; these have been predominantly derived in wealthy settings and not validated through most of the world [4–6]. One intrinsic barrier to researchers’ use of chief complaints, rather than diagnoses, has lain in the absence of standardization of chief-complaint nomenclature, terminology and taxonomy for the recording, translating, and cataloguing of complaints among emergency patients presenting to care in these global settings [7]. The few efforts in this direction that do exist lack the imprimatur and validation standards of an international body [8]. Our group worked with colleagues from the African Federation of Emergency Medicine building on work completed by Rice et al. [3] to create a pilot symptom list for use in low resource settings, representing an attempt by an international criterion-setting body in emergency care to standardize this type of collection of undifferentiated chief complaints in the emergency and acute care setting.

The challenge of translating free-text chief complaints to support syndromic surveillance, operational needs and research work has been extensively addressed in high-income settings [4, 9, 10]. However, and as noted, lists derived in resource-rich settings often have not been validated across national boundaries, nor in health care systems characterized by substantially different patterns of care-seeking, resource-availability, and degrees of development of emergency care. In addition, while chief complaints do not perfectly map to disease burden, a standardized language for recording and analyzing chief complaints allows actors across the spectrum of acute and emergency care, including community, pre-hospital, and hospital-based health-care providers, to effectively communicate and develop system-level priorities based around the signs and symptoms most often experienced by the patients they serve. A straightforward and universal chief complaint list, tested and validated in a global setting, would be of profound benefit to clinicians, researchers, and policy-makers world-wide as it would allow for the use of chief complaint data in the quantification, analysis, and evidence-based planning that emergency care in low-resource settings is urgently in need of.

This paper presents methodological strategy that can be exported to other settings to refine a local chief complaint list. The authors piloted a draft symptom list against traditional free-text chief compliant recording and thereby sought to calibrate and improve the functionality of the pilot symptom list within an exemplar emergency system in South Africa.

## Methods

### Overview

Incorporated as a part of a larger study on human immunodeficiency virus (HIV) testing in Emergency Departments in South Africa, the pilot symptom list (Appendix 1) was used for chief complaint coding in a large multi-center Emergency Department (ED) based observational study in South Africa wherein study staff (predominantly HIV counsellors or nurses, with research training), collected both free text chief complaints and then made a good-faith effort to match chief complaints to one of the pre-determined symptoms from the pilot symptom list [11].

The original prospective observational study, conducted between June 2017 and July 2018, was embedded in the larger Walter Sisulu Infectious Diseases Screening in Emergency Departments (WISE) Study that implemented point-of-care HIV testing in the ED and collected extensive demographic data on ED patients. This study collected data across three EDs in the Eastern Cape Province, South Africa, where each of the three EDs was sampled for a period of 6 weeks. Data was collected on convenience sample of 3357 patients, who enrolled in the WISE study, from across these three hospitals using paper case-report forms (CRFs). For the purposes of this analysis, the free-text chief complaints were then coded using the Medical Dictionary for Regulatory Activities (MedDRA©) nomenclature. Free-text chief complaints were then compared to those identified by study staff using the pilot symptom list and analyzed for clustering using factor analysis. The primary outcome of interest was to assess the adequacy and accuracy of the pilot symptom list in capturing and reflecting patients’ presenting complaints. The secondary outcome of interest was to assess redundancy in the pilot symptom list by observing categories that were never selected or selected significantly frequently. A modified Delphi methodology was used to review the outcomes and observations using these to make recommendations for modifications and amendments to the pilot symptom list.

### Setting

The WISE study was conducted in the Eastern Cape Province in three hospital-based emergency departments. Nelson Mandela Academic Hospital (NMAH) and Mthatha Regional Hospital (MRH) are located in the rural town of Mthatha, and Livingstone Hospital (LH) is in the city of Port Elizabeth. NMAH and LH are both tertiary care centers, they receive referred patients from regional and district hospitals in addition to providing 24-h trauma care. MRH provides 24-h services for walk-in patients and ambulances, while trauma cases are transferred to NMAH. All hospitals maintain 20–50 beds in the ED and are staffed by 1–2 doctors, but are not staffed by physicians or other providers specializing in Emergency Medicine. Patients are seen on a first-come-first-serve basis unless determined to be critically ill or requiring immediate care. Handwritten logbooks and paper medical files are used to track all patients.

### Recruitment and enrolment

Patients presenting for care to the hospital ED during the study period, aged 18 years and older, fully conscious, and clinically stable were eligible for enrollment in the study. Patients who met the inclusion criteria were approached by trained HCT staff as soon as they completed the triage process and were informed of the ongoing study and offered a point-of-care HIV test. Data was also collected on patient demographics, presenting complaint, presenting symptoms, past medical history, and reasons for accepting or declining the HIV test. Written informed consent was sought for all patients. Patients were enrolled 24 h a day throughout the duration of the study.

### Data collection

Data were recorded using CRFs. Responses to *demographic information*, *past medical history,* and *reasons for accepting or declining the HIV test* were recorded using predetermined categorical options or as free text, *presenting complaint* was recorded as free text and later coded using the Medical Dictionary for Regulatory Activities (MedDRA©, MedDRA MSSO, Virginia). *Chief complaints* were recorded using the pilot symptom list. CRFs were scanned and entered using intelligent character recognition (ICR) DataFax software (DataFax©, Clinical DataFax Systems Inc., Hamilton, Ontario, Canada) and centrally double-verified by independent data technicians.

### Data analysis and statistics

Data were analyzed using STATA v.15© (StataCorp, LLC, TX). The pilot symptom list was checked for accuracy against MedDRA-coded chief complaints for each patient. MedDRA-coded chief complaint terms were reviewed by two study team members to determine the frequency of concordance between the MedDRA term and the selected symptom(s) from the pilot symptom list. A ‘match’ was defined as a patient record with a MedDRA term that matched with the symptom(s) selected on the pilot symptom list. A ‘mismatch’ was defined as a patient record with a MedDRA term that was either not present in the pilot symptom list or did not align with the symptom(s) selected on the pilot symptom list. These mismatches were further defined as ‘true mismatches’ aka list errors (when the appropriate symptom(s) matching the MedDRA term did not exist on the pilot symptom list/needed to be added) and ‘false mismatches’ aka rater errors (when the appropriate symptom(s) matching the MedDRA term was available but not selected from the pilot symptom list). Clusters of chief complaints were identified using an exploratory factor analysis of the chief complaint list (48 complaints). Factorability of the chief complaints was determined by inspecting the correlation matrix (correlations > 0.4), the Kaiser-Meyer-Olkin measure of sampling adequacy (KMO > 0.6), and Bartlett’s test of sphericity (*p* < 0.05). The chief complaints were then subjected to factor analysis with an oblique rotation (oblimin), producing as simple a structure as possible while permitting correlations among factors. Factors were retained based on the Scree test (Cattell, 1966). Factor analysis is a statistical data reduction and analysis technique that strives to explain correlations among multiple outcomes as the result of one or more underlying explanations, or “factors.” The technique involves data reduction, as it groups a set of variables based on frequency of concurrence.

### Modified Delphi process

**Fig. 1:**
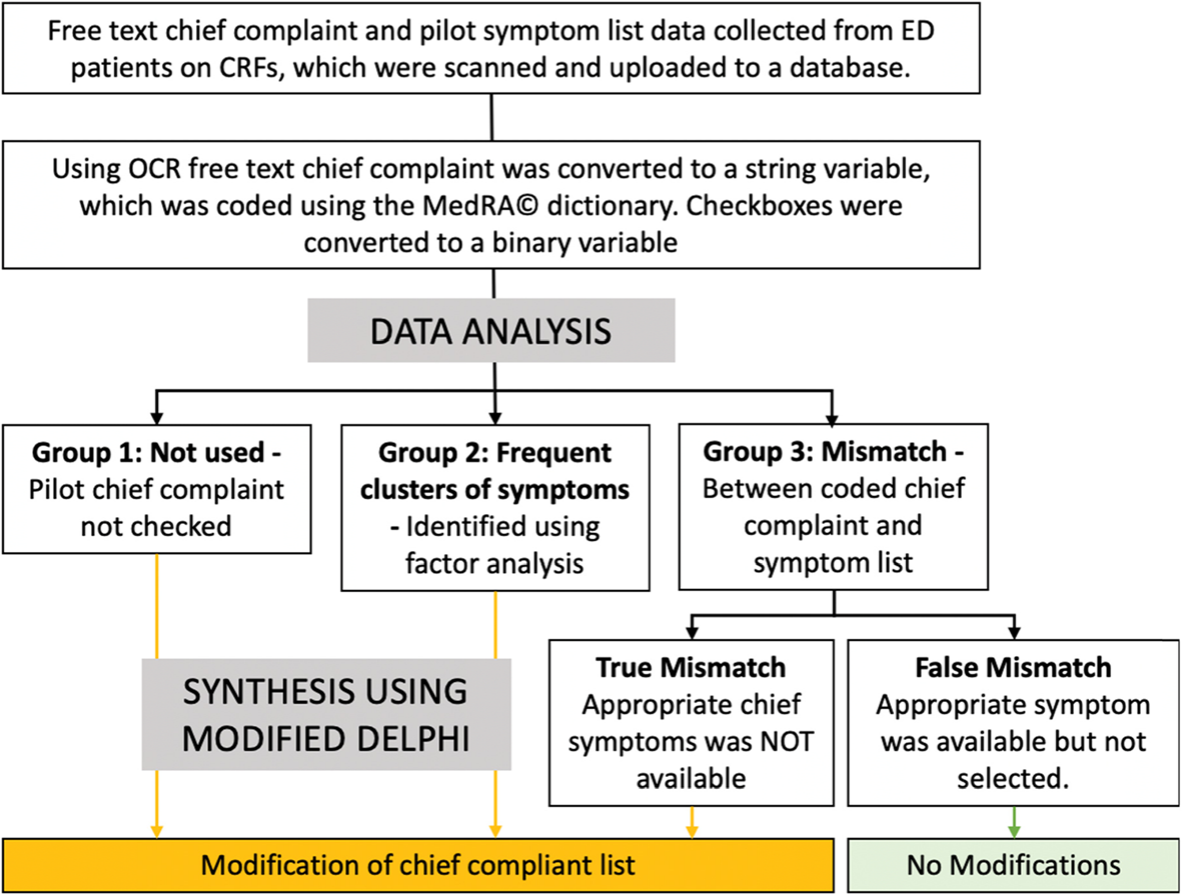
Free-text chief complaint and pilot symptom list tick box matching algorithm

Our algorithm for matching the free-text chief complaint (coded using MedRA ©) and the boxes ticked on the pilot symptom list identified potential chief complaints to be discussed further during a modified Delphi process (Fig. 1).

From this review, final decisions regarding changes to the list were reached systematically using the modified Delphi method. Using this method, each reviewer shared reflections from their independent review, in a round robin fashion, which was recorded and reflected on a whiteboard to the entire group, until no new ideas were forthcoming. Thereafter, reviewers had the opportunity to discuss and clarify each comment/idea shared until group consensus was reached. Notes were kept on rationale for response to each of the mismatches and a detailed review of each of the mismatches, the supporting discussion and resulting recommendations for changes is provided below.

## Results

The intent of this analysis was to address adequacy and functionality of this data set to achieve chief complaint data capture in a real-world resource-constrained emergency medicine setting. There were three distinct activities that were undertaken by the study team to achieve this; firstly, functionality was assessed using staff surveys and feedback via interviews; secondly descriptive data-analysis was used to identify complaints not used, clusters, and mismatches; and thirdly, a modified Delphi approach to discuss observations and recommend changes.

### End-user feedback

Functionality was approached through staff surveys and interviews with the HIV counsellors and nurses who completed the CRFs. These staff reports revealed four key concerns. Firstly, patients and staff struggled with the meaning of some of the terminology used—e.g., “GU” referring to genital or urinary complaints. Secondly, the pilot symptom list was presented as an unordered list—the absence of an interpretable sequence to the list such as alphabetic or body-system based ordering made it difficult for staff to rapidly find complaints. Thirdly, staff reported that they found many of the complaints on the pilot symptom list to be too broad (pain being a primary example) and applied to many patients—this meant that multiple boxes were often ticked. Lastly, staff found it difficult to identify if something was an injury or not, including being unsure, for example, how to classify a patient with an injury 2 weeks ago now presenting for a wound complication. This final concern was raised nearly universally across staff, and we suggest that a major modification to the pilot symptom list should be that the condition/symptom sections include both medical and traumatic chief complaints, and staff should collect further trauma data if an injury is present. In discussions of this topic, staff also reported that they found it easier to think in an ordered fashion about trauma, addressing first mechanism, then location and then intent for injured patients—for example, as in a fall downstairs, causing head injury, occurring due to an assault.

### Descriptive analysis to identified chief complaints for potential modification

The adequacy and accuracy of the pilot symptom list to capture the richness of the data present in the free-text chief complaint fields, ideally in a single category was assessed by undertaking a descriptive analysis of the chief complaints, factor analysis, and matched analysis against the free-text chief complaint coded using the MedRA dictionary.

A total of 3537 patients’ chief complaints were reviewed. Several complaints were selected by staff less than 0.5% of visits and were discussed further, these were foreign body from injury, suspected flu/cold, suspected HIV, blood in urine, confusion/AMS, dental, and ENT. Two categories were never used (1) “suspected malaria” and (2) “foreign body inhaled.” “Pain” was used 2076 times for 58.7% of all patients, therefore was of limited utility in providing discrete additional data. An exploratory factor analysis using the pilot symptom list categories to assess correlation patterns between individual symptoms as selected from the pilot symptom list was completed. Significant chief-complaint clustering was identified with 10 sets of chief complaints that were frequently selected together for the same patient (Table 1).

Based on the match of the chief complaint with the MedRA code for the free-text chief complaint, 640 were identified as ‘potential mismatches’. Of these ‘potential mismatches,’ 191 (29.8%) were confirmed as true mismatches rather than algorithmic errors (i.e., due to coding errors or misreading of data via ICR) when reviewed by the authors. After the Delphi process 6 (3.1%) of the 191 confirmed mismatches were identified as false mismatches aka user errors where an appropriate symptom was available but not checked and 185 (96.9%) were identified as true mismatches aka list errors (Table 2).

**Table 1:** Results of factor analysis groupings

Symptom clustering	Frequency	Discussion
Wound, pain, bleeding from injury	344	All of these are related to penetrating injury, pain does not add new data, not clear if it is beneficial to have both “wound” and “bleeding” from injury
Abdominal pain, pregnancy complication, vaginal bleeding	23	This cluster will likely occur together, perhaps can be addressed with training or changing the location of these complaints
Pain, swelling	421	Most frequently ticked, and add little data to the underlying etiology of the symptoms
Nausea, diarrhoea	66	Likely cluster as often present together, but both need to remain
Cough, chest pain	80	Likely cluster as often present together, but both need to remain
Fever, cold	20	“Cold” does not add additional value
Weakness, shortness of breath	50	Likely cluster as often present together, but both need to remain
ENT, dental	20	Would be possible to merge as likely require similar resources
Blood in urine, GU complaint	30	Most did not understand “GU” complaint, could be addressed by training or remove completely
Abnormal BP, headache	70	Likely cluster as often present together, but both need to remain

**Table 2:** Mismatches identified and resulting recommendations

Chief complaint (***n***)	Discussion	Recommendation
Abdominal pain (22)	Only pain checked; provider did not keep searching for proper CC	Remove pain
Abnormal blood pressure (1)	Only pain checked; provider did not keep searching for proper CC	Change to high blood pressure
Abnormal glucose (2)	Only pain checked; provider did not keep searching for proper CC	Remove pain
Abscess (5)	Pain checked/too general, can be captured in rash/skin problem	Remove abscess
Alcohol/drug problem (2)	Medication issue checked instead of alcohol	Change to poisoning/alcohol/drug problem
Blood in cough/nose (1)	Hemoptysis and epistaxis are separate entities	Change to coughing/vomiting blood, as epistaxis can be captured by ear/nose/mouth
Bloody D/V (4)	Bloody diarrhea deserves its own category to capture dysentery cases; move bloody vomiting to coughing/vomiting blood	Change to bloody diarrhea to capture dysentery
Chest pain (11)	Pain checked; provider did not keep searching for proper CC	Remove pain
Confusion/AMS (1)	AMS is not lay terminology	Change to confusion
Decreased urine output (1)	Pain checked; provider did not keep searching for proper CC	Remove pain
Diarrhea/constipation (2)	Pain checked; provider did not keep searching for proper CC	Remove pain
ENT (12)	ENT is not lay terminology; also, likely unknown term outside of Western medicine	Change to ear/nose/mouth
Eye problem (2)	Issues unrelated to eye problem	Remove abscess
Focal weak/numb (2)	Issues unrelated to focal weak/numb, however can capture stroke-like symptoms in one broad category Limb weakness/facial droop	Change to limb weakness/facial droop
Fracture/deformity (2)	Pain checked; provider did not keep searching for proper CC	No change recommended
Generalized weakness (2)	Pain checked; provider did not keep searching for proper CC	Remove pain
GU complaint (15)	GU is not lay terminology; maybe out of cultural context. Often checked for rectal complaints	Remove GU complaint, add urinary problem, penis/vagina problem or genital problem, and rectal problem
Headache (3)	Occasionally checked in context of traumatic injury	No change recommended
Mass (1)	Not very specific	Change to suspected cancer/mass
Nausea/vomiting (3)	Pain checked; provider did not keep searching for proper CC	Remove pain
Pain (3)	Only three instances where Pain could have better described the intended CC; thousands of instances where pain was checked when another CC could provide more useful data	Remove pain
Pregnancy complication (1)	Complication not lay terminology	Change pregnancy complication to pregnancy problem
Psychiatric illness/SI (1)	Was not checked when it should have been	No change recommended
Rash/skin lesion (4)	Lesion not lay terminology	Change to rash/skin problem
Seizure/convulsion (4)	Convulsion not lay terminology	Change to fits/seizure
Shortness of breath (5)	May be out of cultural context	Change to breathing problem
Speech problem (1)	Was not checked when it should have been	No change recommended
Swelling (2)	Unclear whether refers to generalized edema or focal swelling	No change recommended; will place under limb/swelling heading to discourage use for skin complaints
Syncope/fainting (2)	Syncope not lay terminology	Change syncope/fainting to fainting/dizziness
Vaginal bleeding (2)	Issues unrelated to vaginal bleeding	No change recommended
Generalized weakness	Was not ticked in cases of fatigue or just weakness, thus modified to be more inclusive	Change to generalized weakness/fatigue
GU complaint involved in several mismatches	GU is not lay terminology and was frequently used inappropriately for rectal complaints; likely unknown term outside of Western medicine. Difficult to find a ubiquitous term for these complaints	Change to genital problem
Mass represented < 0.5% of visits	It seems as if trying to capture concern for cancer, but was often not ticked because not explicit enough	Change to suspected cancer/mass
Medication issue represented < 0.5% of visits	Unsure what this is trying to capture	Combine with poisoning/ingestion/medication problem
Wound	Not specific enough and staff did not understand	Change to wound from injury

### Modified delphi

The end-user feedback and descriptive analysis (i.e., low- and-high frequency chief complaints, clusters (Table 1) and mismatches (Table 2) informed the expert-panel modified Delphi analysis and resulted in several substantial changes to the pilot symptom list, as presented in Appendix 2. A total of 10 complaints were removed, 6 were added, and eleven modifications were made. A detailed summary of the observations made and surrounding discussion from the modified Delphi process is available in Appendix 3.

## Discussion

Chief complaints are essential to the practice of emergency care and chief complaint data contains a wealth of information to inform clinicians, researchers, and policy-makers as to the nature and diversity of emergency condition presentations, as well as the emergency care resource and training needs associated with them. As such, the absence of a standard chief complaint naming convention, minimum data set, and organizational strategy that retains functionality in a diversity of settings represents a critical tool gap in global emergency care. By classifying chief complaints, the most challenging portion of the labor of taxonomy is complete. Sorting the chief complaints expressed in the patients’ own words into an established classification system using lay terminology thereby becomes much easier and with this ease comes reliability and consistency, improving data quality and helping fill the data gap in global emergency care.

As described, the absence of a unified, valid, and functional chief complaint short-list with tested utility in global resource-constrained acute care settings has had significant adverse effects on clinical care, research, and informed public. In this paper, we present a systematic approach to refining a chief compliant list for limited resource settings. Themes that were predominant in this analysis included issues of taxonomy, nomenclature, and frequency. Chief complaints that were removed were often taken out because they were overly generic and were selected by staff in preference to more specific and accurate chief complaints available on the list. Elimination of these complaints will improve the frequency with which a specific single accurate complaint is chosen. Inaccurate selection of chief complaint also occurred frequently on the basis of nomenclature confusion. The initial list included United States-centric jargon and abbreviations e.g., “ENT” and “GU” to refer to otolaryngologic and genitourinary complaints, respectively. This naming convention was confusing, opaque, and incomprehensible to the staff completing patient triage as it was not commonly used terminology/nomenclature in that population and culture. The elimination of jargon and acronyms and the use of simpler language/lay terminology to reflect similar sign/symptom complexes address this issue and thereby improve legibility and identification of accurate chief complaints but does not address potential translation errors when used in a non-English-speaking setting.

An additional taxonomic problem identified in factor analysis was the grouping of chief complaints that have substantial overlap (such as pain and swelling) or associate as a part of cardinal presentations of illness (such as chest pain and cough). This was addressed in some cases by combining chief complaints with substantial overlap into a single chief complaint while allowing individual elements of cardinal presentations to remain as standalone complaints using frequency data to inform these changes. Finally, frequency data and mismatch data were used to sub-divide extant chief complaints as well as to include chief complaints not currently captured in the original list of conditions and symptoms but responsible for notable fractions of presentations. One example of this pattern would be “back pain” which was the presenting concern in 2% of all presentations but was not well captured by any existing complaint. This final process and review of the 185 true mismatches resulted in adding 11 new complaints. Examination of frequency data also resulted in the identification of chief complaints with low frequency related to rarity and unlikely to enhance capture rates substantially through inclusion on the chief complaint list, such as “inhaled foreign body.” These latter chief complaints were eliminated from the list.

Strengths of this study include that this is, to our knowledge, the first prospective piloting develop a minimum set of chief complaints intended for use in low- and middle-income settings. One limitation is that establishing this as a minimum set generalizable across geographic and cultural boundaries will require a validation strategy beyond South Africa, including in settings with less mature emergency medical systems and non-English-speaking settings. In recognition of the limitation of testing the pilot symptom list in a single country, the researchers recognize that some of the chief complaints with a low frequency, such as “suspected malaria,” were likely secondary to geographical biases of the database and thus would be beneficial to keep. Additional strengths include the development of a concise 47-item list; the goal of producing a minimum set that will allow for easy aggregation and categorization of patient presentations would not be possible with a more lengthy or more technical list of complaints, such as the Canadian ED Diagnosis Shortlist which captures 99% of all presenting complaints but requires 837 individual chief complaints to do so [10]. The goal of the analysis was to present a rapidly “scan-able” list of complaints that could be used by a triage or check-in provider with little-to-no specialty training in emergency care.

**Fig. 2:**
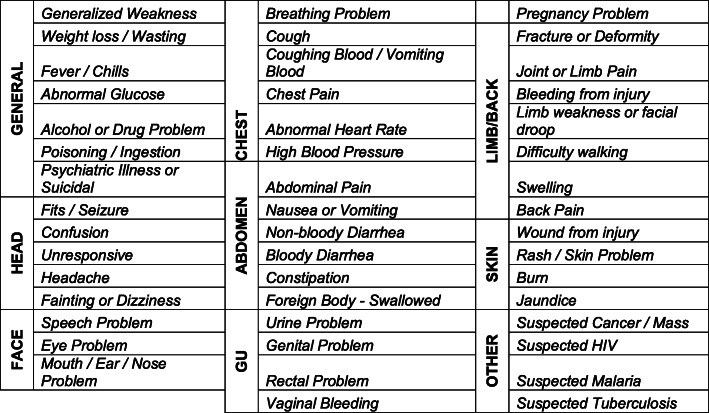
Revised symptom or complaint list

**Fig. 3:**
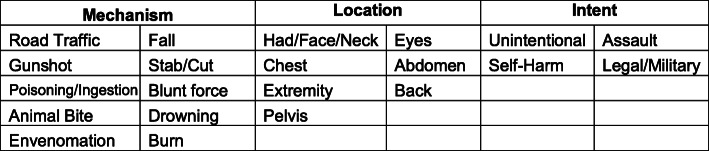
Known injury classification

An additional limitation of this study is that there was no process in place to test the impact of the presentation of the pilot symptom list in its original form, including impact of individual item findability due to ordering effects and overall ease of use. We propose changes to the ordering and organization of the chief complaint list based on functionality data provided by in-depth interviews with study staff. However, testing unique variations of the organization of the pilot symptom list in each of the three sites in addition to interviews about use would have provided more robust data on the impact of user experience effects on functionality of the list overall. Lastly, we used similar data from interviews to inform revisions of the trauma categorization but formal usability testing strategies of these recommendations would add strength to claims of improved functionality. Suggested groupings of the chief complaints by body system for usability follow as Figs. 2 and 3.

## Conclusion

This study presents a systematic methodological sound approach to refine a chief complaint list for low resources settings, namely user feedback, with descriptive analysis to assess frequency of use of the various chief complaints and factor analysis to assess for grouping. Further studies will require validation of suggested recommendations and application of these changes to improve functionality. Additionally, further research will need to determine generalizability of the proposed list across other LMICs outside of South Africa. A patient’s chief complaint is a vital tool, that when easily accessible from a validated list to be wielded by trained emergency medical personnel may help triage patients, streamline emergency care delivery, and improve patient outcomes.

## Figures and Tables

**Fig. 4:**
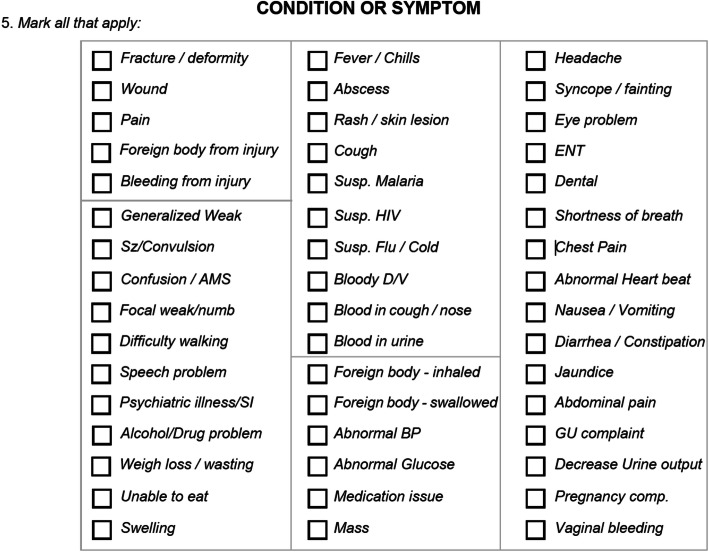
Pilot symptom list

## Data Availability

The anonymized dataset supporting this conclusion will be available upon request to the corresponding author.

## Appendices

### Appendix 2

**Table 3:** Summary of recommended modifications for the pilot symptom list

Added	Removed	Modified	Unmodified
Back pain	Abscess	High blood pressure	Weight loss/wasting
Burn	Bleeding from injury	Abnormal heart rate	Fever/chills
Joint or limb pain (includes swelling)	Foreign body from Injury	Coughing/vomiting blood	Abnormal glucose
Genital problem—complaint of penis, testicle or vulva (includes sexual assault)	Foreign body—Inhaled	Confusion	Headache
Suspected tuberculosis	Pain	Urinary problem	Speech problem
Unresponsive	Suspected flu / cold	Ear/nose/mouth problem	Eye problem
Bloody diarrhea			
Poisoning/ingestion/medication problem	Unable to eat	Non-bloody diarrhea	Cough
Rectal problem	Shortness of breath	Constipation	Chest pain
	Focal weak/numb	Limb weakness/facial droop	Suspected HIV
	Medication Issue	Generalized weakness or fatigue	Suspected malaria
	GU complaint	Alcohol or drug related problem	Abdominal pain
		Fits/seizure	Foreign body—swallowed
		Fainting or dizziness	Nausea or vomiting
		Suspected cancer/mass	Vaginal bleeding
		Pregnancy problem	Fracture or deformity
		Wound from injury	Difficulty walking
		Rash/skin problem	Jaundice
		Breathing problem	Psychiatric illness or suicidal
		Limb/swelling	

### Appendix 3

**Table 4:** Summative review of discussion and recommendations of categories removed, added and changed

Observations (change category)	Discussion	Recommendation
Abscess often misdiagnosed	Abscess is a diagnosis rather than a complaint, associated with rash/skin lesion in group factor analysis	Remove abscess
Bleeding often grouped with wound and pain	Bleeding from injury was associated with wound and pain in group factor analysis, does not contribute helpful information	Remove bleeding from injury
Foreign body from injury represented < 0.5% of visits	Could be captured in “wound from injury”; doesn't affect resource allocation	Remove foreign body from injury
Foreign body inhaled was never checked	Not frequent enough to warrant its own category	Remove foreign body inhaled
Pain was checked in 59% of 3537 visits	Does not provide meaningful information or impact allocation of resources	Remove pain
Suspected flu/cold represented < 0.5% of visits	Does not provide meaningful information or impact allocation of resources	Remove suspected flu/cold
Unable to eat is not specific for any disease process	Could be captured with other general complaints (generalized weakness/fatigue, Weight loss/wasting, etc.)	Remove unable to eat
Suspected HIV represented < 0.5% of visits	Important category for resource allocation/epidemiology	No change recommended
Suspected malaria was never checked	Likely underrepresented in South Africa due to low malaria prevalence	No change recommended
Swelling	Difficult to discern generalized edema vs focal swelling	Place under limb heading to encourage focal use
**Observations** **(add category)**	**Discussion**	**Recommendation**
Back pain not on list	Represented 1.8% of complaints; non-traumatic back pain managed differently than traumatic back pain	Add new category: back pain
Burn	Represented 0.9% of complaints; burns in LMICs have a significant impact on morbidity and mortality, our count is likely not representative of true incidence	Add new category: burn
Joint/MSK pain not on list	Represented about 1.8% of complaints; more specific than pain	Add category: joint or limb pain
Sexual assault is not captured on this form	Sexual assault is a large and under reported problem in LMIC, this is a chance to gain more accurate data	Add new category: sexual assault (under known injury intent, or genital heading)
Suspected tuberculosis not on list	Important category for resource allocation/epidemiology	Add new category: suspected tuberculosis
Unresponsive not on list	Frequently (1.64%) selected in Uganda chief complaint study; distinct entity from confusion/fatigue	Add new category: unresponsive
**Observations** **(change category)**	**Discussion**	**Recommendation**
Abnormal BP	Patients unlikely to present with “low BP” as a chief complaint	Change to high blood pressure
Heart beat	Even though this is low frequency, we suspect that it was underutilized due to poor understanding of “heart beat”	Change to abnormal heart rate
Blood in cough/nose	Epistaxis can be captured by Ear/Nose/Mouth	Change to coughing/vomiting blood
Blood in urine represented < 0.5% of visits,	Likely underrepresented in South Africa due to low schistosomiasis prevalence, too specific	Change to urinary problem
Bloody D/V involved in several mismatches	Bloody diarrhea deserves its own category to capture dysentery cases; move bloody vomiting to coughing/vomiting blood as above	Change to bloody diarrhea
Confusion/AMS represented < 0.5% of visits	“AMS” is not lay terminology	Change to confusion
Decrease urine output	Too specific, can combine with blood in urine	Change to urinary problem
Dental represented < 0.5% of visits	Low frequency complaint, associated with ENT in group factor analysis, thus can merge with ENT	Merge “dental” and "ENT" into "ear/nose/mouth"
Diarrhea/constipation	Diarrhea important cause of morbidity and mortality worldwide, warrants its own category	Split into non-bloody diarrhea and constipation
ENT represented < 0.5% of visits, involved in several mismatches	ENT is not lay terminology; also, likely unknown term outside of Western medicine	Change to ear/nose/mouth
Focal weak/numb was involved in several mismatches	Attempting to capture large strokes with one category	Change to limb weakness/facial droop

## Abbreviations

BOD: Burden of disease
CRF: Case report form
ED: Emergency department
HIV: Human immunodeficiency virus
ICR: Intelligent character recognition
LH: Livingstone Hospital
MRH: Mthatha Regional Hospital
NAMH: Nelson Mandela Academic Hospital
WISE: Walter Sisulu Infectious Diseases Screening in Emergency Departments

## References

[1] Griffey RT, Pines JM, Farley HL, Phelan MP, Beach C, Schuur JD (2015). Chief complaint–based performance measures: a new focus for acute care quality measurement. Ann Emerg Med..

[2] World Health Organization. International statistical classification of diseases and related health problems: 10th revision, Fifth edition. Geneva (CH): World Health Organization; 2016. 131 p. Report No.: 5.

[3] Rice B, Leanza J, Mowafi H, Kamara NT, Mulogo EM, Bisanzo M (2020). Defining high-risk emergency chief compaints: data-driven triage for low- and middle-income countries. Acad Emerg Med..

[4] Aronsky D, Kendall D, Merkley K, James BC, Haug PJ (2001). A Comprehensive set of coded chief complaints for the emergency department. Acad Emerg Med..

[5] Chapman WW, Dowling JN, Wagner MM (2005). Classification of emergency department chief complaints into 7 syndromes: a retrospective analysis of 527,228 patients. Ann Emerg Med..

[6] Horng S, Greenbaum NR, Nathanson LA, McClay JC, Goss FR, Nielson JA (2019). Consensus development of a modern ontology of emergency department presenting problems—the Hierarchical Presenting Problem Ontology (HaPPy). Appl Clin Inform..

[7] Mowafi H, Dworkis D, Bisanzo M, Hansoti B, Seidenberg P, Obermeyer Z, et al. Making recording and analysis of chief complaint a priority for global emergency care research in low-income countries. Acad Emerg Med. 2013; 20(12):1241-1245. https://doi.org/10.111/acem.12262. 10.1111/acem.12262 24283813

[8] Rice BT, Bisanzo M, Maling S, Joseph R, Mowafi H (2018). Derivation and validation of a chief complaint shortlist for unscheduled acute and emergency care in Uganda. BMJ Open..

[9] Haas SW, Travers D, Tintinalli JE, Pollock D, Waller A, Barthell E (2008). Toward vocabulary control for chief complaint. Acad Emerg Med..

[10] Unger B, Afilalo M, Boivin JF, Bullard M, Grafstein E, Schull M (2010). Development of the Canadian emergency department diagnosis shortlist. CJEM..

[11] Hansoti B, Stead D, Parrish A, Reynolds SJ, Redd AD, Whalen MM (2018). HIV testing in a South African Emergency Department: A missed opportunity. PLoS ONE..

